# 131 Incremental yield of adding anterior nares to axilla/groin for Candidozyma auris surveillance among hospitalized patients

**DOI:** 10.1017/ash.2026.10543

**Published:** 2026-06-23

**Authors:** Estera Jachowicz-Matczak, Piotr Heczko, Jacek Czepiel, Mateusz Gajda, Jadwiga Wókowska-Mach

**Affiliations:** 1 Collegium Medicum Jagiellonian University; 2 Jagiellonian Univeristy / Univeristy Hospital in Cracow; 3 Jagiellonian University Medical School

## Abstract

**Background:** Antimicrobial resistance in Clostridioides difficile threatens frontline therapies, particularly vancomycin and metronidazole. Rapid genomic screening can flag putative resistance mechanisms, informing infection prevention and stewardship. We present pilot WGS data focused on vancomycin- and metronidazole-associated determinants. Methods A multicenter collection included the University Hospital in Kraków and five long-term care facilities in Małopolska, in 2022-2023. Sixty-six C. difficile strains were sequenced (WGS, AVITI PE300) and analyzed bioinformatically (Genpax IDEM, version 2.6); eight originated from colonized individuals, the remainder from CDI cases. We extracted the prevalence of vancomycin-associated genes (vanR-Cd, vanS-Cd, vanT-Cd, vanG, vanZ1, and vanS-Cd_T369I variant) and the metronidazole-associated gene nimB-Cd. Results Markers linked to vancomycin resistance were frequent: vanR-Cd (93.9%), vanT-Cd (71.2%), vanS-Cd (63.6%), vanG (59.1%), vanZ1 (51.5%), and vanS-Cd_T369I (4.5%). The metronidazole resistance marker nimB-Cd occurred in 95.5% of isolates. Notably, only one strain lacked any vancomycin resistance gene, and this isolate was recovered from a patient with CDI. These pilot genomic findings suggest widespread carriage of loci associated with reduced susceptibility to vancomycin and metronidazole in our regional C. difficile population. Conclusions In this pilot dataset, C. difficile isolates frequently carry genomic elements associated with vancomycin and metronidazole resistance. However, the mere presence of these markers, such as nimB or genes under vanRS regulation, may not independently indicate phenotypic resistance without specific functional mutations or regulatory changes. Phenotypic confirmation remains essential to establish clinical relevance; standardized susceptibility testing and integration of genotypic–phenotypic correlations with clinical data will follow in the next phase. Until then, these preliminary WGS results should be interpreted with caution and primarily guide prioritization of surveillance and stewardship actions.

